# “I Can’t Get No Satisfaction”—Psychosocial Aspects and Awareness of Negative Impacts in Chemsex Users: Results from an Anonymous Online Survey

**DOI:** 10.3390/brainsci14070666

**Published:** 2024-06-29

**Authors:** Marcus Gertzen, Sinan Karcher, Johanna Schwarz, Cornelia Rosenberger, Moritz Strasburger, Andrea Rabenstein, Anna-Martina Strasser, Ulrich Palm, Tobias Rüther

**Affiliations:** 1 Department of Psychiatry, Psychotherapy, and Psychosomatics, Medical Faculty, University of Augsburg, 86156 Augsburg, Germany; 2 Department of Psychiatry and Psychotherapy, University Hospital Munich, 80336 Munich, Germany; 3 P3 Clinic, 82327 Tutzing, Germany

**Keywords:** sexualized substance use, chemsex, MSM, minority stress, shame, queer identity, sexual self-concept

## Abstract

Chemsex is the interplay of substance use by men who have sex with men (MSM) in sexual contexts. The minority stress model and the identity process theory are explanatory models. In this study, we investigated whether (i) differences in certain psychosocial aspects (i.e., shame, aspects of queer identity, and sexual self-concepts) exist between chemsex users and non-users, and (ii) which factors influence an awareness of negative impacts in chemsex users. We conducted an anonymous, cross-sectional, online survey, including sociodemography, sexual history, history of substance use, validated scales for shame-proneness, aspects of queer identity, and sexual self-concepts. Our analysis comprised descriptive statistics, *t*-tests, Spearman’s correlations, and a multiple linear regression model. We recorded a total of 3257 datasets with 107 chemsex users. Chemsex users showed higher rates for risky sexual behavior. Values for shame proneness, more negative aspects of queer identity, and sexual self-concepts were elevated in chemsex users with an awareness of negative impacts. Sexual anxiety, intravenous substance use, and having had a difficult process coming out were significant predictors of feeling negative impacts. Aspects of shame, queer identity aspects, and sexual self-concepts play an important role in the field of chemsex. Different explanatory models seem to be relevant for different subgroups of chemsex users. Chemsex users with an awareness of a problem were particularly vulnerable and distressed but had the highest motivation for change. Prevention, counseling, and care might profit from the inclusion of these aspects. Further anti-stigma campaigns and a specialization of the healthcare system are needed. Registration: DRKS00022336, date: 29th of October, 2020.

## 1. Introduction

“Chemsex” is a neologism from the two words “chemical” and “sex” [1]. It is defined as the intake of mephedrone, γ-hydroxybutyrate (GHB), γ-butyrolactone (GBL), and methamphetamine (also known as “the four chems”) in order to sustain, enhance, disinhibit, or facilitate sexual experiences with multiple sexual partners, typically in sex sessions lasting several hours or days [2,3] that are mainly performed by men who have sex with men (MSM) [4]. Qualitative studies found that motivators for chemsex may include minimizing subjective risk perception, potentiating pleasurable feelings, building a feeling of intimacy, coping with sexual minority stigma, and boosting confidence, among other factors [5,6,7].

Other authors expanded the definition, including other substances like ketamine or cocaine [8]. Until today—to our knowledge—there is no consensus definition of the term “chemsex” [9].

Data on the prevalence of chemsex are scarce. In a representative, nationwide, American sample, 10.3% of the polled MSM stated that they used chemsex-specific substances in the past 12 months [10]. For a Canadian sample, a prevalence of 21.5% for chemsex among MSM could be detected [11]. One of the biggest prevalence studies was the European MSM Internet Survey (EMIS), which showed a high range of prevalence rates between the different European countries and even urban centers [12], with Brighton having the highest (16.3%) and Sofia the lowest (0.4%) prevalence rates of chemsex among the participants of the survey. But a more recent British prevalence study reported an overall prevalence of chemsex among MSM living in England at 6.6% [13]. In another British cross-sectional study, the prevalence rate for sexualized chemsex substance use within the past year was 6.1% [14]. This emphasizes the temporal and spatial fluctuations of the phenomenon. As a cause for chemsex, stigma and the minority-stress model [15] have been widely discussed, with the internalization of heteronormativity and homonegativity causing feelings of guilt whenever one performs homosexual sex [6]. Affected people may try to counter these feelings of guilt with substance use, resulting in the combination of sexuality and the use of “chems”. According to another construct, the “identity process theory”, gay and bisexual men may experience sexuality-related stressors that can undermine feelings of self-esteem, self-efficacy, continuity, and positive distinctiveness, which can result in threats to identity brought about by these stressors. In response to identity threat, gay and bisexual men may engage in chemsex as a coping response that encompasses and facilitates various largely maladaptive coping strategies and tactics. The more chemsex is perceived as enhancing the identity process and averting identity threat, the more central it is likely to be to the identities of participants. The centrality of chemsex to one’s identity may preclude self-withdrawal from the practice [16]. In both concepts-in the minority stress model as well as in the identity process theory—aspects such as shame, aspects of queer identity, and sexual self-concepts play a role.

Severe risks for physical and mental health have been described for chemsex users. From a somatic point of view, lethal overdoses and sexually transmittable infections (STIs), including the human immunodeficiency virus (HIV) or the Hepatitis C virus, are of high importance [8,17,18]. However, increased odds for the acquisition of bacterial infections, e.g., syphilis, gonorrhea, and chlamydia, among others, could be observed [19,20]. More frequent risky sexual behaviors, like condomless anal intercourse, group sex, high numbers of new sexual partners, or fisting, could be identified [21,22] with an apparent increase in risk-taking behavior alongside an increase in the number of used substances [23], leading to an increased risk for STIs.

Mental disorders are the other issue of high relevance. Chemsex users showed higher rates of mental health symptoms like depression, anxiety, somatization, addiction, and psychotic symptoms. Furthermore, higher incidences of non-consensual sex acts compared with the non-chemsex group could be shown, and symptoms of PTSD were more frequent. For those who practiced the administration of intravenous drugs (referred to as “slamsex”, or “slamming”), the mental health symptoms were more severe [24,25,26,27]. Even higher rates of suicidal ideations and behavior could be evidenced, especially in individuals who carried out slamsex [28]. Therefore, chemsex users are a subgroup of MSM with particular risk factors, substantial sexual risk inequalities [14], and with a need for a holistic therapy approach.

However, data on specialized treatment options are limited. In situations of overdosing, emergency treatment options are essential [18,29]. Concerning HIV, pre-exposure prophylaxis (PreP)—meaning the prophylactic intake of tenofovir and emtricitabine for a significant reduction of infection risks of HIV for MSM having condomless, receptive, anal intercourse [30]—was established as an important harm-reduction tool and—contrary to initial concerns about adherence—was confirmed as an effective prophylactic strategy in chemsex users [31]. A matter of concern in the context of chemsex is the development of resistance to antibiotics in bacterial STIs in general [32].

In cases of addiction or other psychiatric comorbidities, specialized treatment options like withdrawal or general approaches are available. But a holistic chemsex-specific approach is—to our knowledge—lacking at the moment [9]. Therapeutic options often comprise community-engaged responses involving social and cultural strategies of harm reduction and sexual health promotion before, during, and after a chemsex session [33]. One psychotherapeutic manual for group therapy called “getting off” addresses MSM who use methamphetamine [34]. It was shown to be effective in reducing drug use and HIV risk. Sexuality is mentioned, but the complexity of chemsex is not alluded to. The novel psychoactive treatment UK Network (NEPTUNES) published a clinical guideline concerning novel psychoactive substances and club drugs [35]. Aspects of sexuality and the interplay between sexuality and substance use are missing. A new chemsex-specific tool is a harm-reducing app called “budd”, comprising information about chemsex in general, as well as HIV medication, interactions of drugs, a planning tool, a low threshold support service even in the case of an emergency, etc. [36]. One case report demonstrated the disappearance of chemsex behavior after an administration of tDCS [37], but no randomized, controlled trials are available at the moment. Even though specific therapy approaches are on the rise, clinical care is still insufficient [38] and psychotherapeutic options have to be optimized.

In this regard, certain psychosocial aspects, e.g., shame, aspects of queer identity, and sexual self-concepts of queer patients, have not been included and addressed sufficiently in prevention, counseling, and care. Therefore, the inclusion of certain psychosocial aspects might help to increase adherence by increased feelings of inclusion of the affected individuals, which could, in a second step, lead to better rates of harm-reduction outcomes. This would be in line with the identity process theory (see above). Another well-established aspect of psychotherapy is motivation, as it is a proximal determinant of behavior and, consequentially, of behavioral change [39]. As a correlate, an awareness of the problem has already been shown to be an essential base for change motivation in addictive disorders [40,41,42]. Accordingly, promoting problem awareness could lead to increased motivation and adherence to therapy, as well as an improvement in therapy outcomes. Based on these considerations, we formulated the following questions. 

In this study, we tried to investigate whether (i) differences in certain psychosocial aspects exist between chemsex users and non-users, and (ii) which of these factors influence awareness of the problem in chemsex users. Results could help to further optimize prevention, counseling, and care in this field with improved outcomes.

## 2. Materials and Methods

### 2.1. Procedure

A web-based survey tool (LimeSurvey) was used to collect data between December 2020 and June 2021. Using convenience and snowball sampling, participants were recruited through online advertisements and newsletters of queer sports clubs, political organizations, dating platforms, podcasts, leisure groups, community centers, and health centers (list of partners in Appendix A). Accessing the study’s website (www.sexstudie.com), participants received a brief description of the nature of the survey, an estimated completion time, and an assurance of anonymity. Due to strict privacy regulations, no IP addresses were recorded. No incentives were offered for completing the survey, and participation was voluntary. Participants could opt out of the study at any time. After providing their informed consent, participants were asked about (i) demographic variables, (ii) sexual and gender identity, (iii) sexual history in the previous 12 months, (iv) sexualized substance use in the previous 12 months, (v) attitudes towards chemsex (if applicable), (vi) lesbian/gay/bisexual (LGB) identity aspects (if applicable), (vii) shame proneness, and (viii) sexual self-concepts. A seriousness check at the end of the questionnaire allowed participants to exclude their contribution from the analysis. For datasets to be included in the analyses, they had to be (i) completed and (ii) truthfully answered by participants (iii) over 18 years of age who were (iv) sexually active within the last 12 months. This study concept was approved by the ethics committee of Ludwig Maximilians University (LMU) Munich and was part of a larger project examining queer sexualities (DRKS00022336).

### 2.2. Participants

The sample comprised 1307 responses, which were categorized into the following sexual behavior groups based on the gender identity of the respondent and the gender of their sexual partners within the last 12 months: men who had sex with men (MSM), men who exclusively had sex with women (MSW), women who had sex with women (WSW), and women who exclusively had sex with men (WSM). Within each sexual behavior group, participants were further subcategorized based on their sexualized drug-use pattern within the last 12 months. MSM was further subcategorized analogous to this procedure into a “No Chemsex” group and a “Chemsex” group.

### 2.3. Measures

The entire questionnaire was administered in German, and gender-sensitive language was implemented whenever possible.

#### 2.3.1. Sociodemographic Measures

The socio-demographics queried were participants’ age, current self-defined relationship status, the highest level of education, employment status, town size, and migration background.

Participants were asked by which of the following sexual orientation they felt best described: “heterosexual”, “rather heterosexual”, “bisexual”, “rather gay/lesbian”, “gay/lesbian”, “queer”, and “not listed”. They were also presented with a list to choose the sexual identity that best matched theirs, namely: “female (cis)”, “female (trans)”, “male (cis)”, “male (trans)”, “intersex”, “non-binary”, and “not listed/diverse”. A definition of “cis” and “trans” was provided in the instructions.

#### 2.3.2. Sexual History in the Previous 12 Months

Sexual history was assessed using adaptive questioning. Participants indicating that they were sexually active within the last 12 months were asked to specify if this was with a “man (cis/trans)”, “a woman (cis/trans)”, or both. Participants were also asked to estimate the number of sexual partners, condom use, PrEP use, and number of STIs within the last 12 months. Furthermore, HIV status was queried.

#### 2.3.3. Sexualized Substance Use

Adaptive questioning was implemented for assessing substance use. Participants reporting general substance use in the last 12 months (excluding alcohol, caffeine, and nicotine) were shown a list of substances to choose from, including “methamphetamine”, “GHB/GBL”, “mephedrone/cathinone”, and “ketamine”. When indicating the usage of a substance, they were asked to estimate their consumption frequency (“yearly”, “quarterly”, “monthly”, “weekly”, and “daily”) and the ratio of sexualized usage for this substance. Sexualized use was rated on a five-point Likert scale ranging from “Never in conjunction with sex” to “Always in conjunction with sex”. They were also asked if they had injected one of these substances within the last 12 months.

#### 2.3.4. Attitudes toward Sexualized Substance Use

Participants who used one of the substances (methamphetamine, GHB/GBL, mephedrone/cathinone, ketamine) in conjunction with sex were asked about their attitudes toward sexualized substance use. (i) “Negative Impact”—“Within the past 12 months, have you felt that the use of mind-altering substances in a sexual context (chemsex) has negatively affected your life”? (ii) “Wish to Reduce”—“Within the past 12 months, have you had the desire to use fewer or no more mind-altering substances in a sexual context (chemsex)”? If participants indicated “No” for “Wish to Reduce”, they were not asked about (iii) “Need for Support”—“Have you had the desire to seek professional support for this within the last 12 months”? These statements were rated on a five-point Likert scale, ranging from “Yes” to “Rather yes”, “Unsure”, “Rather no”, and “No”.

#### 2.3.5. Shame Proneness

To measure shame proneness, we used the German version of SHAME [43], a self-report tool comprising 21 items to address three dimensions of shame. Participants were presented with hypothetical scenarios examining one of the following facets of shame: “cognitive” (seven items), “bodily” (seven items), and “existential” (seven items). One such scenario to rate how ashamed a participant would feel is: “I address someone, who I really should know, with the wrong name” [44]. The rating occurred on a five-point Likert scale, ranging from “0 = not at all” to “4 = extremely”. The subscales were averaged to provide a final score for shame proneness, with higher scores indicating higher shame proneness. The internal consistency was good with Cronbach’s alpha for shame proneness *α* = 0.84.

#### 2.3.6. LGB Identity Aspects

The German version of the “Lesbian, Gay, and Bisexual Identity Scale” (LGBIS) [45] is a 27-item self-report instrument to assess eight aspects important to LGB identity. It is an adaptation of the English version of the “Lesbian, Gay, and Bisexual Identity Scale” [46]. Subscales include “Acceptance Concern” (three items; *α* = 0.79), “Concealment Motivation” (three items; *α* = 0.82), “Identity Uncertainty” (four items; *α* = 0.77), “Internalized Homonegativity” (three items; *α* = 0.86), “Difficult Process” (three items; *α* = 0.83), “Identity Superiority” (three items; *α* = 0.71), “Identity Affirmation” (three items; *α* = 0.81), and “Identity Centrality” (five items; *α* = 0.82). Subscale definitions by the original authors can be found in Appendix B. Using adaptive questioning, the LGBIS was presented only to participants who indicated same-sex sexual behavior within the last 12 months. Participants were free not to answer the LGBIS if they felt the wording (“schwul/lesbisch” = “gay/lesbian”) was incompatible with their sexual orientation. Statements such as “I think a lot about how my sexual orientation affects the way people see me” were rated for approval on a six-point Likert scale, ranging from “1 = Disagree strongly” to “6 = Agree strongly”. The items were averaged for each subscale; higher scores indicated a higher expression of the measured dimension.

#### 2.3.7. Sexual Self-Concepts

For assessing sexual self-concepts, participants filled out the ”Multidimensionale Fragebogen zur Sexualität” (MFS) [47]. It is the German adaptation of the “Multidimensional Sexuality Questionnaire” (MSQ) [48]. The MFS is a self-rating tool totaling 60 items divided into 12 subscales, each representing a sexual self-concept. Dimensions covered are “Sexual Esteem” (*α* = 0.89), “Sexual Preoccupation” (*α* = 0.90), “Internal Sexual Control” (*α* = 0.61), “Sexual Consciousness” (*α* = 0.55), “Sexual Motivation” (*α* = 0.85), “Sexual Anxiety” (*α* = 0.89), “Sexual Assertiveness” (*α* = 0.84), “Sexual Depression” (*α* = 0.87), “External Sexual Control” (*α* = 0.74), “Self-Monitoring” (*α* = 0.74), “Fear of Sex” (*α* = 0.82), and “Sexual Satisfaction” (*α* = 0.90). Participants were asked to provide tendencies on statements such as “I’m constantly thinking about having sex” on a five-point Likert scale, ranging from “1 = Not at all characteristic of me” to “5 = Very characteristic of me”. Items were again averaged for each subscale, with higher scores corresponding to higher amounts of the sexual self-concept. Lacking sufficient internal consistency, the subscales “Internal Sexual Control” and “Sexual-Consciousness” were excluded from further analyses.

#### 2.3.8. Seriousness Check

At the end of the survey, participants were asked to indicate how truthfully they answered the survey (“How truthfully did you fill out the questionnaire”?) on a five-point Likert scale, ranging from “1 = Truthfully” to “5 = Not truthfully”. This allowed respondents to exclude their submissions from the study, thus increasing data quality [49]. Only participants indicating that they answered “Truthfully” or “Rather Truthfully” were considered for analyses.

### 2.4. Analytic Plan

Statistical analyses were performed using SPSS version 28. Only respondents meeting all inclusion criteria were considered for the final analyses. Participants were assigned a group based on their gender identity and sexual behavior (MSM, MSW, WSW, WSM). Participants who could not be assigned to one of these four groups were excluded from further analyses. The remaining respondents were subcategorized by their sexualized drug use pattern into a “No Chemsex” group and a “Chemsex” group. Participants were assigned to the “Chemsex” group if they indicated consumption of at least one substance as “Always in conjunction with sex” or “Rather in conjunction with sex”. Participants not consuming any substances or consuming them “Never in conjunction with sex” were assigned to the “No Chemsex” group. Participants showing a mixed-use pattern were excluded from further analyses. To better distinguish between chemsex behavior in general and problematic chemsex behavior, another subgroup (“Negative Impact”) was created based on participants feeling negative consequences of their chemsex behavior (“Yes”/“Rather yes”). The overall frequency of sexualized substance use (“Chemsex Frequency”) was determined by the substance with the highest frequency of use where participants indicated using “Always in conjunction with sex” or “Rather in conjunction with sex”. Descriptives were analyzed for sociodemographic variables, gender and sexual identity, sexual history, prevalence and frequency of chemsex, and attitudes toward chemsex. Psychometric measurement instruments were included if their Cronbach’s internal reliability exceeded *α* ≥ 0.70. Comparisons between the “Chemsex” and “No Chemsex” groups, as well as between the “Negative Impact” and “No Chemsex” groups, were performed for the SHAME and the subscales of the LGBIS and MFS using *t*-tests. To measure the effect size of the difference between the groups, Cohen’s *d* was calculated. For the analyses performed, we carried out a Bonferroni correction based on multiple testing (MSF, SHAME, LGBIS) with a significance level of *p* < 0.05/3 = 0.0167. Correlations were calculated for (1) “Negative Impact” and (2) “Chemsex Frequency” with different variables. Depending on the level of measurement, correlative effect sizes were exploratively calculated using Spearman’s correlation or transformation of *z*-values from the Mann–Whitney U-test to *r* [50]. Finally, a multiple linear regression model (method = stepwise) was used to determine the predictive value of non-psychometric variables, sexualized substance use patterns, shame proneness, LGB identity, and sexual self-concepts on “Negative Impact” in the “Chemsex” group. All variables significantly correlated with “Negative Impact” and “Chemsex Frequency” were included in the model. Correlative effect sizes were classified as “medium” for *r* = 0.20–0.30 and as large for *r* > 0.30 [51,52]. All data presented in this study will be made available upon reasonable request.

## 3. Results

### 3.1. Chemsex by Sexual Behavior

A total of *n* = 3257 datasets could be recorded, with *n* = 1392 responses meeting our inclusion criteria, with a dropout rate of 59.8%. *n* = 1307 could be categorized into one of the following sexual behavior groups: “MSM” (*n* = 781), “MSW” (*n* = 131), “WSW” (*n* = 118), and “WSM” (*n* = 277). Participants not grouped stated they were “non-binary” (*n* = 57), “intersex” (*n* = 2), “not listed/diverse” (*n* = 9), or that they did not have sex with someone of a binary gender identity (*n* = 17). For reasons of practicability, responses were further subcategorized by their sexualized substance use behavior into “Chemsex” (*n* = 114), “No Chemsex” (*n* = 1160), and “Mixed-Use” (*n* = 33), even though the definition of the term “chemsex” comprises only MSM. A detailed summary of participants’ sexualized substance use behavior can be found in Table 1. A majority of participants (59.8%) defined themselves as MSM of whom 107 participants (13.7%) reported engaging in chemsex. 

### 3.2. Sociodemographics

Due to the low number of non-MSM indicating “chemsex behavior”, and due to the original definition of chemsex, all further analyses were performed within the MSM subgroup (*n* = 759). The groups “No Chemsex” (*n* = 652) and “Chemsex” (*n* = 107) were independent. “Negative Impact” (*n* = 35) was defined as a subgroup of the “Chemsex” group. Table 2 shows a detailed summary of respondents’ gender identity, age, level of education, employment status, town size, and migration background. A majority of the non-chemsex users (98.3%) and chemsex users (99.1%) defined themselves as cis male. Also, 52.5% of the individuals in the non-chemsex group were aged between 30 and 49, and 79% of the chemsex group had the same age. The percentage of upper education level was high (29.7% for non-chemsex users and 19.6% for chemsex-users), and a majority of participants worked full time (62.9% for non-chemsex users and 57.9% for chemsex users). The rate of full-time employees for the subgroup of chemsex users who felt a negative impact of chemsex in their lives was lower, with 48.6%. Interestingly a majority of chemsex users lived in a metropolis (50.5%), whereas only a minority of non-chemsex users reported living in a city of the same size (26.4%). The majority of both subgroups stated that they had no migration background (80.7% of the non-users and 71% of the chemsex users).

### 3.3. Sexual History

A description of the sexual history of the “No Chemsex”, “Chemsex”, and “Negative Impact” groups can be found in Table 3. Results reported are for sexual identity, current relationship status, number of sexual partners, condom use, PrEP use, acquirement of STIs, and HIV status. Participants could choose not to disclose their HIV status. A majority of 85.3% of the non-chemsex users described themselves as gay or rather gay, whereas 90.7% of the chemsex users stated the same. Concerning relationship status, 56.4% of the non-chemsex users and 61.7% of the chemsex users reported being in a relationship. Interestingly, a lower rate of 51.4% of the chemsex users who stated they felt a negative impact on their life by chemsex reported being in a relationship. The number of sexual partners and risky sexual behavior was higher for chemsex users (e.g., 44.8% of the chemsex users stated that they never use a condom, whereas 29.9% of the non-users stated the same). For STIs, 23.4% of the chemsex users reported having had multiple STIs before, and only 2.6% of the non-users reported the same. Interestingly, the rate of multiple STIs in the subgroup of chemsex users that stated chemsex has had a negative impact on their lives was higher, with 42.9% of the individuals responding that way. PreP usage was higher in the chemsex group, with 43% for the chemsex users and only 14.1% for the non-users. Concerning HIV status, 39.3% of the chemsex users stated that they did not know about their HIV status, whereas only 9.5% of the non-chemsex group stated the same. For the subgroup of chemsex users with an awareness of the problem, 45.7% of the participants reported being HIV positive, whereas only 2.8% of the total chemsex group and 10.1% of the non-chemsex group reported the same. No participant of the chemsex user subgroup with awareness of the problem stated that he did not know about his HIV status.

### 3.4. Chemsex Frequency and Attitudes

Chemsex frequency was determined by the substance with the highest frequency of use, where participants indicated predominantly sexualized use. Participants not indicating a wish to reduce their chemsex behavior were not asked about their need for support. A detailed summary of chemsex frequency and attitudes toward chemsex for the “Chemsex” and “Negative Impact” groups can be found in Table 4. Participants answering the question if they felt a negative impact of chemsex on their lives with “yes” or “rather yes” were included in the subgroup with an awareness of the problem. A majority of 67.2% reported being involved in chemsex quarterly or monthly. No participant reported daily use. Still, the quarterly and monthly use of chemsex substances was pronounced (74.2%) in the subgroup of chemsex users who felt a negative impact of chemsex on their lives. Also, 57.2% of the negative impact subgroup stated they had a wish to reduce substance use, and even 39.4% of them pointed out a need for support, while only 23.4% of the chemsex users without an awareness of the problem stated they had a wish to reduce substance use, and 16.9% of them asserted a need for support.

### 3.5. Shame Proneness, LGB Identity, and Sexual Self-Concepts Comparison

For shame proneness, no significant difference in mean values between the “No Chemsex” (*M* = 1.724, *SD* = 0.596) and “Chemsex” groups was found (*M* = 1.705, *SD* = 0.586); *t*(757) = 0.30, *p* = 0.763; *d* = 0.031.

When comparing the “Negative Impact” group with the “No Chemsex” group on shame proneness, the “Negative Impact” group (*M* = 2.008, *SD* = 0.621) showed significantly higher values than the “No Chemsex” group (*M* = 1.724, *SD* = 0.596); *t*(685) = −2.74, *p* = 0.006; *d* = −0.476.

LGB identity is comprised of eight subscales. T-tests revealed significant differences for three of them in the total chemsex user group. The “No Chemsex” group (*M* = 3.428, *SD* = 1.386) showed significantly more concealment motivation than the “Chemsex” group (*M* = 2.838, *SD* = 1.376); *t*(755) = 4.08, *p* < 0.001; *d* = 0.426. Furthermore, the “Chemsex” (*M* = 2.698, *SD* = 1.059) group reported significantly more identity superiority than the “No Chemsex” group (*M* = 2.095, *SD* = 1.032); *t*(755) = −5.57, *p* < 0.001; *d* = −0.582. Identity centrality was significantly more pronounced in the “Chemsex” group (*M* = 4.153, *SD* = 1.138) than in the “No Chemsex” group (*M* = 3.767, *SD* = 1.181); *t*(755) = −3.15, *p* = 0.002; *d* = −0.329. 

Identity uncertainty showed higher values in the “No Chemsex” group (*M* = 1.631, *SD* = 0.936) than in the “Chemsex” group (*M* = 1.449, *SD* = 0.800); *t*(157.8) = 2.13, *p* = 0.035; *d* = 0.199, which was not significant for reasons of Bonferroni correction (*p* < 0.0167). The same was true for the comparison of the “No Chemsex” (*M* = 3.109, *SD* = 1.336) group and the “Chemsex” group in terms of acceptance concerns (*M* = 2.779, *SD* = 1.364); *t*(755) = 2.36, *p* = 0.018; *d* = 0.247. Identity affirmation was more pronounced in the “Chemsex” group (*M* = 4.38, *SD* = 1.316) than in the “No Chemsex” group (*M* = 4.109, *SD* = 1.268); *t*(755) = −2.04, *p* = 0.042; *d* = −0.212, but values were not significant. Most interestingly, insignificant results for mean differences were returned for internalized homonegativity (*p* = 0.148; *d* = 0.151) and having had a difficult process coming out (*p* = 0.469; *d* = 0.076).

For the “Negative Impact” group, significant differences in mean values were found for two out of eight of the subscales of the LGBIS. Identity superiority was significantly higher in the “Negative Impact” group (*M* = 2.714, *SD* = 0.901) than in the “No Chemsex” group (*M* = 2.095, *SD* = 1.032); *t*(683) = −3.48, *p* < 0.001; *d* = −0.603. The “Negative Impact” group (*M* = 4.095, *SD* = 1.369) also described a significantly more difficult process of coming out than the “No Chemsex” group (*M* = 3.363, *SD* = 1.422); *t*(683) = −2.97, *p* = 0.003; *d* = −0.516. 

Insignificant results were returned for concealment motivation (*p* = 0.69; *d* = −0.069), identity uncertainty (*p* = 0.184; *d* = −0.231), acceptance concerns (*p* = 0.477; *d* = −0.124), and identity affirmation (*p* = 0.45; *d* = 0.131). Furthermore, the “Negative Impact” group (*M* = 2.286, *SD* = 1.435) reported higher scores for internalized homonegativity than the “No Chemsex” group (*M* = 1.76, *SD* = 1.091); *t*(36.15) = −2.14, *p* = 0.04; *d* = −0.474, and for identity centrality (*M* = 4.246, *SD* = 1.048, *M* = 3.767, *SD* = 1.181); *t*(683) = −2.35, *p* = 0.019; *d* = −0.408, but values were not significant for reasons of Bonferroni correction.

For sexual self-concepts, *t*-tests reported significant differences for two of the 10 subscales in the total chemsex user group. The “Chemsex” group (*M* = 2.785, *SD* = 0.96) showed significantly more sexual esteem than the “No Chemsex” group (*M* = 2.419, *SD* = 0.868); *t*(757) = −3.98, *p* < 0.001; *d* = −0.415. The “Chemsex” group (*M* = 1.391, *SD* = 0.899) reported significantly more self-monitoring than the “No Chemsex” group (*M* = 1.146, *SD* = 0.8); *t*(757) = −2.88, *p* = 0.004; *d* = −0.301.

Insignificant results were returned for sexual preoccupation (*p* = 0.728; *d* = −0.036), sexual motivation (*p* = 0.357; *d* = −0.096), sexual assertiveness (*p* = 0.101; *d* = −0.171), sexual depression (*p* = 0.847; *d* = 0.02), external sexual control (*p* = 0.095; *d* = −0.175), fear of sex (*p* = 0.415; *d* = 0.085), and sexual satisfaction (*p* = 0.061; *d* = −0.196). Sexual anxiety was emphasized in the “Chemsex” group (*M* = 1.08, *SD* = 0.997) in comparison to the “No Chemsex” group (*M* = 0.882, *SD* = 0.864); *t*(757) = −2.15, *p* = 0.032; *d* = −0.225, but values were not significant for reasons of Bonferroni correction.

For the “Negative Impact” group, significant differences were found for five out of 10 sexual self-concepts. Sexual anxiety was significantly more pronounced in the “Negative Impact” group (*M* = 1.869, *SD* = 1.056) than in the “No Chemsex” group (*M* = 0.882, *SD* = 0.864); *t*(36.48) = −5.43, *p* < 0.001; *d* = −1.128. The “Negative Impact” group (*M* = 1.731, *SD* = 1.105) showed significantly more sexual depression than the “No Chemsex” group (*M* = 1.064, *SD* = 0.94); *t*(685) = −4.05, *p* < 0.001; *d* = −0.703. Also, self-monitoring was significantly stronger in the “Negative Impact” group (*M* = 1.811, *SD* = 1.057) than in the “No Chemsex” group (*M* = 1.146, *SD* = 0.801); *t*(36.12) = −3.67, *p* < 0.001; *d* = −0.817. Fear of sex was significantly more present in the “Negative Impact” group (*M* = 1.851, *SD* = 1.168) than in the “No Chemsex” group (*M* = 1.263, *SD* = 0.943); *t*(36.42) = −2.93, *p* = 0.006; *d* = −0.617. The “Negative Impact” group (*M* = 1.897, *SD* = 1.129) reported significantly lower sexual satisfaction than the “No Chemsex” group (*M* = 2.32, *SD* = 0.983); *t*(685) = 2.46, *p* = 0.014; *d* = 0.427.

Insignificant differences were returned for sexual esteem (*p* = 0.83; *d* = 0.047), sexual preoccupation (*p* = 0.914; *d* = 0.019), sexual motivation (*p* = 0.155; *d* = 0.247), and external sexual control (*p* = 0.459; *d* = −0.129). Sexual assertiveness was lower in the “Negative Impact” group (*M* = 2.057, *SD* = 1.05) than in the “No Chemsex” group (*M* = 2.436, *SD* = 0.915); *t*(685) = 2.365, *p* = 0.018; *d* = 0.410, but values were not significant for reasons of Bonferroni correction.

### 3.6. Negative Impact and Chemsex Frequency Correlations

Correlations between the feeling of chemsex negatively affecting one’s life and sociodemographic and sexual history variables were computed using Spearman’s correlation or the transformation of *z*-values to *r*, depending on the variable’s level of measurement. All responses classified into the “Chemsex” group were included in these analyses. The same procedure was applied to correlations of overall chemsex frequency.

For “Negative Impact”, no significant correlations were found for the sociodemographic variables of age (*r*(105) = −0.003, *p* = 0.973), level of education (*r*(105) = 0.018, *p* = 0.855), town size (*r*(105) = 0.044, *p* = 0.655), and having a migration background (*r*(105) = 0.174, *p* = 0.072). Between “Negative Impact” and variables concerning the sexual history of respondents, only acquirements of STI showed a significant positive correlation (*r*(105) = 0.233, *p* = 0.016), with more STIs increasing the feeling of chemsex harming one’s life. Neither being in a relationship (*r*(105) = −0.105, *p*= 0.279), nor condom usage (*r*(105) = −0.087, *p* = 0.375), nor PrEP usage (*r*(105) = 0.001, *p* = 0.991), nor the number of sexual partners (*r*(105) = 0.101, *p* = 0.299) had any significant correlation with the “Negative Impact” variable. While “Negative Impact” did not show any significant correlation with “Chemsex Frequency” (*r*(105) = 0.099, *p* = 0.312), having injected one of “the four chems” in the previous 12 months did significantly correlate with “Negative Impact” (*r*(105) = 0.268, *p* = 0.006). This means that having injected one of these substances in the past 12 months correlated significantly with an increased feeling of chemsex harming one’s life.

For “Chemsex Frequency”, none of the sociodemographic variables, including age (*r*(105) = 0.003, *p* = 0.975), level of education (*r*(105) = 0.139, *p* = 0.153), town size (*r*(105) = 0.055, *p* = 0.574), and having a migration background (*r*(105) = 0.127, *p* = 0.190) showed significant correlations. Of the variables concerning the sexual history of respondents, only the number of sexual partners showed a significant positive correlation with “Chemsex Frequency” (*r*(105) = 0.222, *p* = 0.022), with a higher chemsex frequency correlating with more sexual partners. Neither being in a relationship (*r*(105) = −0.097, *p* = 0.317), nor condom (*r*(105) = −0.109, *p* = 0.265) and PrEP usage (*r*(105) = 0.167, *p* = 0.085), nor the number of STIs acquired (*r*(105) = 0.134, *p* = 0.170) showed significant correlations with the frequency of chemsex a person reported. Just as for “Negative Impact”, having injected ketamine, GHB/GBL, mephedrone, or methamphetamine significantly correlated with an increased report of chemsex (*r*(105) = 0.244, *p* = 0.012).

### 3.7. Shame Proneness, LGB Identity, and Sexual Self-Concept Correlations

Correlations between the feeling of chemsex negatively impacting one’s life and shame proneness, LGB identity, and sexual self-concepts were computed using Spearman’s correlation. All responses classified into the “Chemsex” group were included in these analyses. The same procedure was applied to correlations of overall chemsex frequency. All large effect sizes (*r* > 0.30) were highly significant (*p* < 0.001).

Shame proneness showed a highly significant positive correlation with “Negative Impact” (*r* = 0.35), which means that higher levels of shame proneness correlated with a stronger feeling of negative influence of chemsex on one’s life.

Concerning aspects of LGB identity, analyses found that higher values of internalized homonegativity (*r* = 0.385) and having had a more difficult process coming out (*r* = 0.319) were significantly correlated with feeling more negative impacts caused by one’s chemsex habit.

While reporting more sexual anxiety (*r* = 0.505), sexual depression (*r* = 0.458), and fear of sex (*r* = 0.405) significantly correlated with a stronger feeling of chemsex negatively affecting one’s life, the opposite was found for sexual satisfaction (*r* = −0.404). This means that feeling more harmed by one’s chemsex habit correlated with lower levels of sexual satisfaction.

No significant correlations were found between “Chemsex Frequency” and shame proneness, LGB identity, or sexual self-concepts. A full display of all correlation coefficients can be found in Table 5.

### 3.8. Prediction of Negative Impacts

Multiple regression analysis was performed (method = stepwise) to evaluate which variables could help predict the feeling of chemsex negatively impacting one’s life.

Included independent variables for MSM were shame proneness, concealment motivation, internalized homonegativity, identity uncertainty, acceptance concerns, identity affirmation, a difficult process coming out, sexual motivation, sexual anxiety, sexual assertiveness, sexual depression, self-monitoring, fear of sex, sexual satisfaction, intravenous substance use, the acquirement of STIs, and chemsex frequency. The model constructed was highly significant and explained 37.3% of the variance (*F* = (3, 103) = 22.04; *p* < 0.001). Sexual anxiety (*β* = 0.454, *p* < 0.001), intravenous substance use (*β* = 0.235, *p* = 0.003), and having had a difficult process coming out (*β* = 0.209, *p* = 0.013) were significant predictors of feeling negative impacts caused by chemsex. In this model, increased sexual anxiety, having had a difficult coming out process, and having injected one of “the four chems” in the past 12 months were good indicators for an increased subjective report of negative consequences of one’s chemsex habit.

## 4. Discussion

In this anonymous online study, we tried to investigate whether differences in certain psychosocial aspects (i.e., aspects of shame, queer identity aspects, and sexual self-concepts) between chemsex users and non-users existed and which factors influence the awareness of the problem in chemsex users. Thus, the aim was to contribute aspects to further optimize prevention, counseling, and care of chemsex users.

The results of our study concerning sexual identity and sexual orientation showed that MSM was mainly affected by chemsex. This is in line with earlier studies, which could already define MSM as the main group of concern in terms of this phenomenon [53]. The sociodemographic data of our participants revealed that mainly middle-aged MSM with an upper educational level, full-time employment, mainly an absence of a migration background, and a residence in a metropolis was affected by chemsex in our study. While these sociodemographic factors have also been found in previous research examining chemsex users, they remain solely descriptive, and causality can only be speculated upon at this moment [12,38,54]. However, they should be examined in future research projects.

Concerning risky sexual behaviors and prevention measures, the number of sexual partners was higher for chemsex users than for non-users. Return rates for condom usage in chemsex users were lower, and rates for PreP usage were higher. Accordingly, a significantly higher rate of a history of STIs could be demonstrated in chemsex users. This statement also corresponds to works from previous literature [22,23,55]. Interestingly, rates for a diagnosis of HIV infection were low in our chemsex group. At the same time, this was not true for the subgroup of chemsex users who felt that chemsex had a negative impact on their lives. Here, we could show elevated rates for HIV infections, which is in line with earlier studies again [14]. HIV infection itself might play a role in the attribution of chemsex being seen as problematic by the individuals being affected. This would correspond well to our finding, that higher rates of STIs correlate with more awareness of a problem. Still, we have to emphasize that 39.3% of the total chemsex user sample stated that they did not know about their HIV status, in contrast to 0% of the chemsex users who saw chemsex as problematic. Therefore, the rate in the total chemsex user sample might have been much higher, which illustrates particular challenges for prevention campaigns in this field. However, we can summarize that the subgroup of chemsex users who stated that chemsex had a negative impact on their lives showed lower employment levels, lower rates of being in a relationship, and higher rates of HIV infections than the total chemsex user sample and controls, which might contribute to the awareness of chemsex being problematic. With regard to chemsex frequency and attitudes, we saw comparable frequencies between chemsex users and the subgroup of affected individuals with awareness of a problem. Therefore, a clear wish to reduce substance use and a need for support was pointed out by the participants of the study who saw that chemsex had a negative impact on their lives in contrast to those who did not. A wish for more information and advice centers for chemsex users by the affected could already be shown in earlier studies [38], but we could demonstrate different stages of motivation in this study.

In terms of shame proneness, we could not observe clear significant differences between the total chemsex user group and controls. Still, this was not true for the subgroup of chemsex users who saw chemsex as being problematic. We could observe significantly higher mean values of shame proneness for chemsex users with an awareness of a problem in comparison to the controls. Shame could already be demonstrated as an important factor in addiction [56] and always has been connected to sexuality. In the context of chemsex, substance use meeting sexual activities in MSM might even reinforce feelings of shame. Therefore, in our eyes, it is important to address feelings of shame when counseling chemsex users.

Concerning LGB identity, higher mean values for identity superiority and identity centrality could be found in the total chemsex group when compared to controls. Furthermore, the no-chemsex group showed more concealment motivation than the total chemsex user sample. Interestingly, insignificant results for mean differences were returned for internalized homonegativity and having had a difficult process coming out. Because of the Bonferroni correction, higher values for identity uncertainty and acceptance concerns in controls were not significant, as were higher values for identity affirmation in the total chemsex user sample.

This leads us to the conclusion that the classic minority stress model [15] should not serve as an etiological explanatory model for all chemsex users equally. However, our findings fit well within the identity process theory [16], which considers identity aspects and the integration of chemsex behavior as a safeguarding strategy of one’s identity in identity-threatening circumstances. Accordingly, this might explain higher mean values of more positive perceived identity aspects and chemsex of the total chemsex sample. Nevertheless, a different picture emerged for the subgroup of chemsex users with awareness of the problem.

Identity superiority was significantly higher in the negative impact group than in the “No Chemsex” group. The negative impact group also described a significantly more difficult process of coming out than the no-chemsex group. Higher values for internalized homonegativity could be detected in the subgroup of chemsex users with awareness of a problem as for controls, but these were not significant due to the Bonferroni correction. Still, one could see these results as a trend because it just missed the corrected significance level with *p* = 0.04. For identity centrality, analysis returned higher values for the negative impact group than for the no-chemsex group, but results were not significant also due to the Bonferroni correction. In this context, the link to feelings of shame of the subgroup of chemsex users who saw their chemsex behavior as problematic is of specific importance because this could be based on the concept of internalized homonegativity. This shows, in our eyes, the continued importance of the minority stress model. Hence, a combination of the minority stress model and the identity process theory could be an explanation of the results. Accordingly, on the basis of internalization processes, negative social consequences could be attributed to chemsex earlier, with feelings of guilt and shame serving as promoters for perceiving chemsex as problematic. In earlier stages, chemsex might have been perceived as identity-stabilizing—a feeling that precludes the individual from letting go of chemsex habits. We, therefore, might see different stages of a continuum in the development of awareness in our study, with some chemsex users perceiving more positive effects of identity stabilization according to the identity process theory at an earlier stage of their motivational journey, and some chemsex users perceiving more negative social and emotional effects according to the minority stress model in a more advanced stage. In the past, an increasing awareness of the problem with increasing disease severity has already been demonstrated for other addictions, such as alcohol addiction [57]. Still this result could also be caused by the cross-sectional design of the study, which represents different, inter-individual manifestations of a characteristic at one single measuring point. Furthermore, we did not detect the period of time for which chemsex had already been performed beyond the 12-month interval mentioned. In summary, this result should be assessed with caution, and further studies are needed to clarify this instance. In order to understand the psychosocial aspects underlying chemsex even better, future research may also examine the cost–benefit analyses individuals perform when engaging in this behavior according to social exchange theory [58] as it might provide a framework for understanding how individuals continue engaging in harmful behaviors due to perceived immediate benefits [59]. Understanding these internal considerations may facilitate better prevention, counseling, and support strategies tailored to the motivations and experiences of chemsex users.

For sexual self-concepts, higher values for sexual esteem could be found in chemsex users when compared to controls. This corresponds well to higher values for identity superiority and identity centrality. Also here, the identity process theory [16] could serve as an explanatory model. At the same time, more self-monitoring could be detected in the chemsex user group. Interestingly, previous findings could show a higher tendency for somatization in chemsex users [27]. Accordingly, a higher tendency to self-observation could serve as an explanation for a higher tendency to somatization.

For chemsex users who were aware of negative impacts, higher values could be detected for sexual anxiety, sexual depression, self-monitoring, and fear of sex. Lower values could be shown for sexual satisfaction. Insignificant differences could be detected for sexual assertiveness. One explanation for higher mean values of these negative aspects, and the attribution of a negative impact on the lives of chemsex users, could be higher rates of STIs and having injected in the same group. This indicates more mental and somatic issues, which is very well in line with the previous literature, particularly in terms of psychosis, addiction, suicidal crisis, and the acquisition of HIV and Hepatitis C [21,26,60,61]. Accordingly, the finding that sexual anxiety, intravenous substance use, and having had a difficult process coming out predicted if chemsex was perceived as problematic seems to be in line with the other findings of our study. However, we were unable to show at what point exactly chemsex users associate their pattern of use with negative consequences. This is again due to the cross-sectional design of the study, and longitudinal studies are needed to further clarify this issue. On the other hand, minority stress itself could also explain a higher awareness of the problem, as negative consequences could have been attributed primarily to homosexual acts. Accordingly, this raises the question of the direction of the effect relationship, which unfortunately could not be clarified in this cross-sectional design. It also shows the need for further longitudinal study designs. 

One of the strengths of this study is that—to our knowledge—certain psychosocial aspects, like shame, aspects of queer identity, and sexual self-concepts of chemsex users have been studied for the first time. In addition, the great importance of awareness of a problem was demonstrated in a reasonably large sample. These aspects appear to be particularly important for prevention, counseling, and care in this field.

Limitations include the anonymity of the survey. We were, therefore, unable to rule out theoretical multiple participation. Snowball sampling is also a limitation, which is why the data cannot be assumed to be representative of the general population. Multiple stigmatizations could also only be represented to a limited extent with our questionnaire, and possible additive effects should be the subject of future research. In addition, the cross-sectional design of the study should be mentioned, which is why no statements could be made about causality. Furthermore, the questionnaire was relatively long, which is why particularly motivated participants were overrepresented in this dataset due to the exclusion criteria. The long questionnaire also explained the relatively high drop-out rate. The high rate of queer institutions and sexual health centers where the study was advertised may also have influenced rates of STIs and attitudes towards homosexuality. Furthermore, it should be emphasized that the results relate to a specific subcultural subgroup of MSM, which must be considered when interpreting the results. For this reason, further representative studies of the general population are urgently needed. In addition, the data were collected during the SARS-CoV-2 pandemic, which could have biased the results.

## 5. Conclusions

In summary, we showed that aspects of shame, queer identity aspects, and sexual self-concepts play an important role in the field of chemsex. Furthermore, explanatory models like the minority stress model and the identity process theory seem to play a role in different subgroups of chemsex users without any of these models being able to explain all facets of the phenomenon. The subgroup of chemsex users with an awareness of a problem appeared to be particularly vulnerable and distressed, but at the same time, they showed the highest motivation for change. Consequently, the topics of stigma, identity, shame, and self-concept should be particularly addressed in a counseling and therapeutic setting. Problem awareness should also be explored in detail accordingly. These findings are the main results of our study.

The results could underline the relevance of stigmatization and experiences of rejection in this field, which is why anti-stigma campaigns and specialized treatment of the affected is of high relevance. Particularly in the area of chemsex, interdisciplinary care for patients should be sought whereby practitioners should be trained in dealing with shame, minority stress, and queer identity aspects. Prevention campaigns should also be further expanded. In general, the health system should be provided with more resources specifically in relation to sexual health and the mental health of minorities.

Future studies may profit from the inclusion of the previously mentioned aspects, and more studies with longitudinal design are needed to clarify causality and effect relationships. Representative studies on the topic of chemsex in the general population are urgently needed to further minimize risk of bias since this study used a snowball sampling approach with a survey exclusively in the German language, which might have biased the results.

## Figures and Tables

**Table 1:** Chemsex behavior by sexual behavior.

	MSM		MSW		WSW		WSM	
	*n*	%	*n*	%	*n*	%	*n*	%
**Total**	781	59.8	131	10.0	118	9.0	277	21.2
**Chemsex Behavior**								
Chemsex	107	13.7	5	3.8	0	0.0	2	0.7
No Chemsex	652	83.5	123	93.9	115	97.5	270	97.5
Mixed-Use	22	2.8	3	2.3	3	2.5	5	1.8

Note. Total *n* = 1307. Groups based on sexual behavior in past 12 months. MSM = men who had sex with men, MSW = men who exclusively had sex with women, WSW = women who had sex with women, WSM = women who exclusively had sex with men.

**Table 2:** Sociodemographics of respondents (MSM) by chemsex behavior.

	No Chemsex		Chemsex		Negative Impact	
	*n*	%	*n*	%	*n*	%
**Total**	652		107		35	
**Gender**						
Male (cis)	641	98.3	106	99.1	35	100
Male (trans)	11	1.7	1	0.9		
**Age**						
18–19	16	2.5				
20–24	70	10.7	3	2.8	2	5.7
25–29	83	12.7	9	8.4	1	2.9
30–39	195	29.9	44	41.1	15	42.9
40–49	147	22.6	33	30.9	10	28.5
50–59	114	17.5	15	14	6	17.1
60–69	21	3.2	3	2.8	1	2.9
70 and Older	6	0.9				
**Education**						
Secondary School						
None	2	0.3				
Lower	18	2.8	2	1.9		
Intermediate	53	8.1	11	10.3	4	11.4
Upper	194	29.7	21	19.6	9	25.7
Apprenticeship	86	13.2	16	15	5	14.3
University						
Bachelor	73	11.2	12	11.2	4	11.4
Master	187	28.7	41	38.3	12	34.3
Doctorate/PhD	39	6	4	3.7	1	2.9
**Employment**						
Full-time	410	62.9	62	57.9	17	48.6
Part-time	54	8.3	20	18.7	7	20
Self-Employed	58	8.9	14	13.1	5	14.3
Student	81	12.4	5	4.7	3	8.5
Retired (regular)	13	2	1	0.9	1	2.9
Retired (early)	17	2.6	3	2.8	2	5.7
Not employed	19	2.9	2	1.9		
**Residency**						
Rural (<10 k)	96	14.7	7	6.5	2	5.7
Small city (10–100 k)	150	23	14	13.1	6	17.1
Mid-sized city (100–500 k)	150	23	15	14	6	17.1
Big city (500 k–1 m)	84	12.9	17	15.9	5	14.3
Metropolis (>1 m)	172	26.4	54	50.5	16	45.8
**Migration Background**						
Yes	126	19.3	31	29	13	37.1
No	526	80.7	76	71	22	62.9

Note. Total *n* = 759. Groups based on chemsex behavior and attitude in past 12 months. “No Chemsex” and “Chemsex” are independent groups. “Negative Impact” is a subgroup of “Chemsex”. Only MSM was included.

**Table 3:** Sexual history of respondents (MSM) by chemsex behavior.

	No Chemsex		Chemsex		Negative Impact	
	*n*	%	*n*	%	*n*	%
**Total**	652		107		35	
**Sexuality**						
Gay/Lesbian	476	73.0	86	80.4	24	68.6
Rather Gay/Lesbian	80	12.3	11	10.3	6	17.1
Bisexual	71	10.9	8	7.5	4	11.4
Rather Heterosexual	10	1.5	1	0.9		
Heterosexual	3	0.5				
Queer	10	1.5	1	0.9	1	2.9
Not listed	2	0.3				
**Relationship**						
Yes	368	56.4	66	61.7	18	51.4
No	284	43.6	41	38.3	17	48.6
**Sexual Partners ^a^**						
1	135	20.7	3	2.8		
2–5	270	41.4	17	15.9	6	17.1
6–11	133	20.4	21	19.6	4	11.4
12–30	76	11.7	32	29.9	11	31.5
31–50	20	3.1	14	13.1	7	20
51–100	12	1.8	14	13.1	6	17.1
101 and More	6	0.9	6	5.6	1	2.9
**Condom Use ^a^**						
Never	195	29.9	48	44.8	15	42.9
Rather not	150	23	43	40.2	16	45.7
50/50	77	11.8	6	5.6	2	5.7
Rather	100	15.3	8	7.5	2	5.7
Always	130	20	2	1.9		
**PrEP Use ^a^**						
Never	510	78.2	50	46.7	16	45.7
Rather not	17	2.6	2	1.9	1	2.9
50/50	13	2	5	4.7	1	2.9
Rather	20	3.1	4	3.7	2	5.7
Always	92	14.1	46	43	15	42.8
**STI ^a^**						
None	565	86.7	52	48.6	13	37.1
One	70	10.7	30	28	7	20
Multiple	17	2.6	25	23.4	15	42.9
**HIV ^b^**						
Negative	514	80.4	62	57.9	19	54.3
Positive	61	9.5	42	39.3	16	45.7
Don’t Know	64	10.1	3	2.8		

Note. Total *n* = 759. Groups based on chemsex behavior and attitude in past 12 months. “No Chemsex” and “Chemsex” are independent groups. “Negative Impact” is a subgroup of “Chemsex”. PrEP = Pre-exposure prophylaxis, STI = Sexually transmitted infection. ^a^ Within last 12 months^, b^ Respondents could choose not to answer. Only MSM was included.

**Table 4:** Chemsex frequency and attitudes.

	Chemsex		Negative Impact	
	*n*	%	*n*	%
**Total**	107		35	
**Chemsex Frequency** ^a^				
Yearly	19	17.8	5	14.3
Quarterly	36	33.6	13	37.1
Monthly	36	33.6	13	37.1
Weekly	16	15	4	11.5
Daily				
**Chemsex Attitudes** ^a^				
Negative Impact				
No	36	33.6		
Rather no	22	20.5		
Ambivalent	14	13.1		
Rather yes	16	15	16	45.7
Yes	19	17.8	19	54.3
Wish to Reduce				
No	30	28	2	5.7
Rather no	18	16.8	2	5.7
Ambivalent	17	15.9	5	14.3
Rather yes	17	15.9	6	17.1
Yes	25	23.4	20	57.2
Need for Support				
No	38	49.3	9	27.2
Rather no	16	20.8	5	15.2
Ambivalent	3	3.9	1	3
Rather yes	7	9.1	5	15.2
Yes	13	16.9	13	39.4

Note. Total *n* = 107. Groups based on chemsex behavior and attitude in past 12 months. “Negative Impact” is a subgroup of “Chemsex”. ^a^ Within last 12 months. Only MSM was included.

**Table 5:** Correlations for “Negative Impact” and “Chemsex Frequency”.

	Negative Impact	Chemsex Frequency
**Chemsex**		
Negative Impact		0.099
Chemsex Frequency	0.099	
**Shame Proneness**	0.351 ***	0.08
**LGB Identity**		
Concealment Motivation	0.257 **	0.059
Internalized Homonegativity	0.385 ***	−0.069
Identity Uncertainty	0.29 **	−0.013
Acceptance Concerns	0.256 **	−0.091
Identity Affirmation	−0.246 *	0.066
Identity Superiority	0.112	0.115
Identity Centrality	0.073	0.023
Difficult Process	0.319 ***	−0.163
**Sexual Self-Concepts**		
Sexual-Esteem	−0.151	0.074
Sexual-Preoccupation	−0.031	−0.016
Sexual-Motivation	−0.227 *	0.064
Sexual-Anxiety	0.505 ***	0.031
Sexual-Assertiveness	−0.247 *	0.072
Sexual Depression	0.458 ***	−0.086
External-Sexual Control	−0.07	−0.124
Self-Monitoring	0.295 **	0.096
Fear-of-Sex	0.405 ***	−0.066
Sexual-Satisfaction	−0.404 ***	0.152

Note. *n* = 107. Values displayed are Spearman’s *ρ.* * *p* < 0.05, ** *p* < 0.01, *** *p* < 0.001.

## Data Availability

Due to strict privacy regulations, our research data can be shared only upon reasonable request.

## Abbreviations

EMIS: European MSM internet study, GBL: γ-butyrolactone, GHB: γ-hydroxybutyrate, HIV: human immunodeficiency virus, MSM: men who have sex with men, NEPTUNES: Novel psychoactive treatment UK Network, STIs: sexually transmittable infections.

## Appendix A

**Table A1:** List of recruitment partners.

Name
Abseitz
Aidshilfe Berlin
Aidshilfe Frankfurt
Aidshilfe Kassel
Aidshilfe München
Akademie Waldschlösschen
Andersraum
AStA Hamburg-Queerreferat
Bezirkskrankenhaus Augsburg
CSD Augsburg
CSD Magdeburg
DAV GOC
Deutsche Aidshilfe
DGPPN Queer-Referat
Evangelische Hochschule Berlin
German Rainbow Golfers
Hochschule Merseburg
Jetzt.de
KLuST
Kokon
LESARION
LeZ
LFCD Dresden
LMU Klinikum München
LMU München-Queer-Referat
LSVD Bayern
LSVD Deutschland
LSVD NRW
Maincheck
Man*Check
Projekt 100% Mensch
Queer im Schloss
Queerbeet
Queerflexiv
Queerpride Würzburg
Queerwandern
RadioSUB
ROMEO
Schwulenberatung Berlin
Schwunguntia
Stadt Mannheim-LSBTI-Beauftragung
Stadt München-Koordinierungsstelle Gleichstellung LGBTIQ
Stadt.Land.Schwul
SUB München
Team München
VC Phönix Düsseldorf
Vielbunt

Note. List of the cooperation partners of our study.

## Appendix B

**Table A2:** Subscale definitions.

	LGB Identity
Subscale	Definition
Acceptance Concern	Concern with being stigmatized as a nonheterosexual person.
Concealment Motivation	Concern with and the motivation to protect one’s privacy as a nonheterosexual person.
Identity Uncertainty	Uncertainty about one’s sexual identity.
Internalized Homonegativity	One’s rejection of one’s nonheterosexual identity.
Difficult Process	Belief that one’s nonheterosexual identity development process was difficult.
Identity Superiority	View of favoring nonheterosexual people over heterosexual people.
Identity Affirmation	Affirmation of one’s sexual-minority identity.
	**Sexual Self-Concepts**
**Subscale**	**Definition**
Sexual Esteem	Generalized tendency to positively evaluate one’s capacity to relate sexuality with another person.
Sexual Preoccupation	Tendency to become absorbed in, obsessed with, and engrossed with thoughts about the sexual aspects of life.
Sexual Motivation	Desire to be involved in a sexual relationship.
Sexual Anxiety	Tendency to feel tension, discomfort, and anxiety about the sexual aspects of one’s life.
Sexual Assertiveness	Tendency to be assertive about the sexual aspects of one’s life.
Sexual Depression	Tendency to feel depressed about the sexual aspects of one’s life.
External Sexual Control	Belief that human sexuality is determined by influences outside of one’s personal control (e.g., chance).
Self-Monitoring	Tendency to be aware of the public impression that one’s sexuality makes on others.
Fear of Sex	Fear of engaging in sexual relations with another individual.
Sexual Satisfaction	Tendency to be highly satisfied with the sexual.

Note. Definition of subscales as stated in the validation papers of the German adaptations [45,46,47,48].
